# MRTX-500 Phase 2 Trial: Sitravatinib With Nivolumab in Patients With Nonsquamous NSCLC Progressing On or After Checkpoint Inhibitor Therapy or Chemotherapy

**DOI:** 10.1016/j.jtho.2023.02.016

**Published:** 2023-02-24

**Authors:** Kai He, David Berz, Shirish M. Gadgeel, Wade T. Iams, Debora S. Bruno, Collin M. Blakely, Alexander I. Spira, Manish R. Patel, David M. Waterhouse, Donald A. Richards, Anthony Pham, Robert Jotte, David S. Hong, Edward B. Garon, Anne Traynor, Peter Olson, Lisa Latven, Xiaohong Yan, Ronald Shazer, Ticiana A. Leal

**Affiliations:** a Comprehensive Cancer Center, Pelotonia Institute for Immuno-Oncology, The Ohio State University, Columbus, Ohio; b Department of Cellular Therapeutics, Beverly Hills Cancer Center, Beverly Hills, California; c Current Affiliation: Valkyrie Clinical Trials, Los Angeles, California; d Henry Ford Cancer Institute, Henry Ford Health System, Detroit, Michigan; e Vanderbilt-Ingram Cancer Center, Vanderbilt University, Nashville, Tennessee; f University Hospitals Seidman Cancer Center, Case Comprehensive Cancer Center, Case Western Reserve University, Cleveland, Ohio; g Department of Medicine, University of California San Francisco, San Francisco, California; h Helen Diller Family Comprehensive Cancer Center, University of California San Francisco, San Francisco, California; i Virginia Cancer Specialists, Fairfax, Virginia; j US Oncology Network, The Woodlands, Texas; k Division Of Hematology, Oncology and Transplantation, University of Minnesota Masonic Cancer Center, Minneapolis, Minnesota; l Department of Clinical Research, Oncology Hematology Care, Cincinnati, Ohio; m Current affiliation: Dana-Farber/Brigham and Women’s Cancer Center at Milford Regional Medical Center, Milford, Massachusetts; n Texas Oncology, Tyler, Texas; o Northwest Cancer Specialists, Tigard, Oregon; p Rocky Mountain Cancer Centers, Denver, Colorado; q MD Anderson Cancer Center, The University of Texas, Houston, Texas; r Department Of Medicine, Division of Hematology/Oncology, David Geffen School of Medicine at the University of California Los Angeles, Los Angeles, California; s Winship Cancer Institute, Emory University, Atlanta, Georgia; t University of Wisconsin Carbone Cancer Center, Madison, Wisconsin; u Mirati Therapeutics, Inc., San Diego, California; v Current Affiliation: Department of Hematology and Oncology, Winship Cancer Institute, Emory University, Atlanta, Georgia

**Keywords:** NSCLC, Tyrosine kinase inhibitor, Sitravatinib, Nivolumab, Antitumor activity

## Abstract

**Introduction:**

Sitravatinib, a receptor tyrosine kinase inhibitor targeting TYRO3, AXL, MERTK receptors, and vascular epithelial growth factor receptor 2, can shift the tumor microenvironment toward an immunostimulatory state. Combining sitravatinib with checkpoint inhibitors (CPIs) may augment antitumor activity.

**Methods:**

The phase 2 MRTX-500 study evaluated sitravatinib (120 mg daily) with nivolumab (every 2 or 4 wk) in patients with advanced nonsquamous NSCLC who progressed on or after previous CPI (CPI-experienced) or chemotherapy (CPI-naive). CPI-experienced patients had a previous clinical benefit (PCB) (complete response, partial response, or stable disease for at least 12 weeks then disease progression) or no PCB (NPCB) from CPI. The primary end point was objective response rate (ORR); secondary objectives included safety and secondary efficacy end points.

**Results:**

Overall, 124 CPI-experienced (NPCB, n = 35; PCB, n = 89) and 32 CPI-naive patients were treated. Investigator-assessed ORR was 11.4% in patients with NPCB, 16.9% with PCB, and 25.0% in CPI-naive. The median progression-free survival was 3.7, 5.6, and 7.1 months with NPCB, PCB, and CPI-naive, respectively; the median overall survival was 7.9 and 13.6 months with NPCB and PCB, respectively (not reached in CPI-naive patients; median follow-up 20.4 mo). Overall, (N = 156), any grade treatment-related adverse events (TRAEs) occurred in 93.6%; grade 3/4 in 58.3%. One grade 5 TRAE occurred in a CPI-naive patient. TRAEs led to treatment discontinuation in 14.1% and dose reduction or interruption in 42.9%. Biomarker analyses supported an immunostimulatory mechanism of action.

**Conclusions:**

Sitravatinib with nivolumab had a manageable safety profile. Although ORR was not met, this combination exhibited antitumor activity and encouraged survival in CPI-experienced patients with nonsquamous NSCLC.

## Introduction

Checkpoint inhibitor (CPI) therapy has changed the treatment landscape for NSCLC. Inhibition of immune checkpoints, either as inhibition of programmed cell death protein-1 (PD-1) or its ligand programmed death-ligand 1 (PD-L1) alone, dual checkpoint inhibition in combination with anti–CTLA-4 therapy or in combination with chemotherapy, promotes an antitumor response in patients with NSCLC, exhibiting considerable clinical benefit and improved overall survival (OS) versus chemotherapy alone.^1–11^ Despite these advances, not all patients respond to CPI (primary resistance), and most who benefit initially develop resistance and experience disease progression (acquired resistance).^12^ For patients with CPI-resistant disease, whose treatment options are limited to conventional chemotherapies, there is a need to develop therapeutics to overcome this resistance.

CPI resistance is associated with various mechanisms, including defects in antigen processing, neoantigen loss, abnormal interferon-gamma signaling, coinhibitory checkpoints, and an immunosuppressive tumor microenvironment (TME).^13^ Regulatory T cells (Tregs), myeloid-derived suppressor cells (MDSCs), and M2-polarized macrophages contribute to the immunosuppressive TME by suppressing pro-inflammatory T-cell responses and producing immunosuppressive cytokines.^14^ The abundance of these cells in the TME is regulated by a network of signaling pathways, and novel approaches are under investigation to target the TME, restore antitumor responses, and improve CPI efficacy.^14,15^ Sitravatinib (MGCD516), a receptor tyrosine kinase inhibitor (TKI), targets TYRO3, AXL, MERTK (TAM) receptors, and vascular endothelial growth factor receptor 2 (VEGFR2).^16–18^ TAM receptors have been implicated in tumor progression by functioning as negative immune regulators that dampen the activation of innate immune cells, thereby promoting an immunosuppressive TME. TAM signaling in cancer can facilitate the immune escape of malignant cells by decreasing natural killer-cell antitumor responses, reducing inflammation, and increasing the ratio of M2/M1 macrophages.^16,19^ Given that M2 macrophages have tumor-promoting capabilities involving immunosuppression, angiogenesis, and neovascularization, their increased number versus the immunostimulatory M1 macrophages can promote a protumor TME.^20^ Similarly, VEGFR2-driven angiogenesis stimulates the expansion of immunosuppressive cells including Tregs and MDSCs.^21^ Therefore, targeting TAM receptors and VEGFR2 using sitravatinib may shift the TME toward a less immunosuppressive state and resensitize tumors to CPI.

In preclinical murine models, sitravatinib exhibited potent antitumor activity by targeting the TME, resulting in immune cell changes that enhanced the efficacy of PD-1 blockade, including in a CPI-resistant lung cancer model.^17^ In a first-in-human phase 1/1b study, sitravatinib monotherapy did not have clinically meaningful antitumor activity in patients with advanced NSCLC.^22^ However, in the phase 1 SNOW window-of-opportunity trial in patients with oral cavity cancer, sitravatinib plus nivolumab (PD-1 inhibitor) was well tolerated and led to clinical and pathologic responses.^23^ The study found that sitravatinib monotherapy resulted in a less immunosuppressive TME and was associated with macrophage repolarization toward the M1 type in patients who responded to sitravatinib with nivolumab.^23,24^

We report the final safety and efficacy results from MRTX-500 (NCT02954991), a phase 2 study of sitravatinib with nivolumab in patients with advanced or metastatic nonsquamous (NSQ) NSCLC with disease progression on or after prior CPI (CPI-experienced) or chemotherapy (CPI-naive). This combination was evaluated in NSQ NSCLC owing to a better understanding of the safety profile of TKIs and antiangiogenic agents in this population at the time of study initiation; moreover, sitravatinib plus a CPI combination is being evaluated in squamous NSCLC in ongoing studies (NCT03666143, NCT04921358).^25,26^

## Materials and Methods

### Study Design and Treatments

MRTX-500 was an open-label, parallel, phase 2 study evaluating sitravatinib with nivolumab in patients with advanced or metastatic NSQ NSCLC who were CPI-experienced or CPI-naive (progression on or after platinum-based doublet chemotherapy). CPI-experienced patients were assessed according to the outcome of previous CPI: prior clinical benefit (PCB) (investigator-assessed and Response Evaluation Criteria in Solid Tumors version 1.1 [RECIST v1.1]–defined complete response [CR], partial response [PR], or stable disease for ≥12 weeks followed by radiographic progression of the disease) versus no PCB (NPCB) (investigator-assessed radiographic progression of disease ≤12 weeks after initiation of treatment). CPI-naive patients were assessed according to PD-L1 status: no or low PD-L1 expression (<5% positivity in tumor cells) versus high PD-L1 expression (≥5% positivity) (see Supplementary Appendix for further information on how PD-L1 expression was assessed).

In the phase 1/1b 516–001 trial in patients with advanced solid tumors,^22^ the maximum tolerated dose for sitravatinib was identified as 150 mg once daily. The present study was designed according to the modified toxicity probability interval method;^27^ it was planned to begin with a dose escalation evaluation of two sitravatinib doses (120 and 150 mg, free base formulation) with nivolumab in CPI-experienced patients. Sitravatinib capsules (120 mg) were administered orally once daily in a continuous regimen of 28-day cycles. Nivolumab was administered at fixed dosing as per the package insert at 240 mg every 2 weeks or 480 mg every 4 weeks by intravenous infusion over approximately 30 minutes. Intrapatient changes to the nivolumab dosing schedule were permitted. Treatment was discontinued in case of objective disease progression, unacceptable toxicity, or other reasons, including patient withdrawal, global deterioration of health, loss to follow-up, or death.

This study was approved by the institutional review board and conducted in accordance with the International Conference on Harmonisation Good Clinical Practice guidelines^28^ and the Declaration of Helsinki.^29^ All patients provided written informed consent.

### Patients

Eligible patients were 18 years or older with histologically confirmed advanced or metastatic NSQ NSCLC, measurable disease per RECIST v1.1, and Eastern Cooperative Oncology Group performance status of 0 to 2. Patients had received at least one previous treatment and had progressed on or after this treatment; for CPI-experienced patients, the most recent line of therapy included PD-1 or PD-L1 inhibitors; CPI-naive patients had received previous platinum-based doublet chemotherapy. Concurrent or previous platinum-based chemotherapy was allowed for CPI-experienced patients.

Key exclusion criteria included: uncontrolled brain metastases; history of tumors testing positive for EGFR, ROS1, ALK mutations, ALK fusions, or any other well-characterized driver mutations (e.g., RET fusion, TRK alterations, BRAF mutations); unacceptable toxicity on previous CPI; or active or previously documented autoimmune disease or immunocompromising conditions.

### Study Objectives and Assessments

The primary objective was to assess the clinical activity of sitravatinib with nivolumab in patients with NSQ NSCLC. Clinical activity was evaluated by investigator-assessed objective response rate (ORR) per RECIST v1.1 in the total analysis population of each cohort. Secondary objectives were to evaluate the safety and tolerability of the combination, and secondary efficacy end points including the following: (1) duration of response (DOR); (2) clinical benefit rate (CBR), defined as the percentage of patients with confirmed CR, PR, or stable disease during ≥1 on-study assessment and including ≥6 wk on-study; (3) progression-free survival (PFS); (4) OS; and (5) 1-year survival rate. All responses were confirmed in subsequent scans per RECIST v1.1.

Exploratory objectives included assessing the effect of the combination on circulating PD-L1, immune cell populations, and cytokines, and on tumor cell PD-L1 expression, tumor-infiltrating immune cells, and gene expression signatures. Further information on-study assessments and biomarker methods are available in the Supplementary Appendix.

### Statistical Analyses

**Figure 1.**
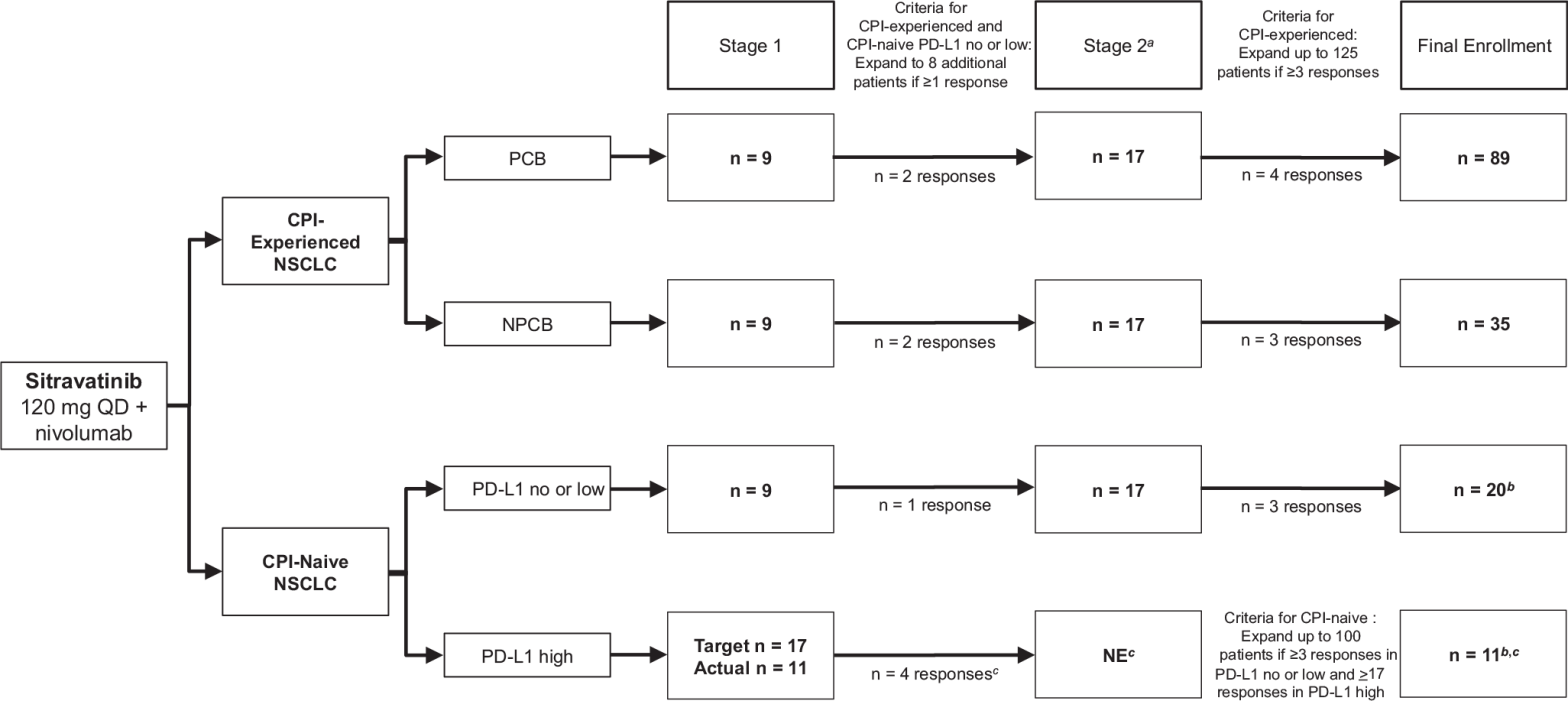
Study diagram of the two-stage enrollment process and responses observed for CPI-experienced and CPI-naive patients with nonsquamous NSCLC. ^*a*^Patient numbers in stage 2 include those carried forward from stage 1. ^*b*^Enrollment in the CPI-naive cohort was stopped owing to changes in the treatment landscape. One CPI-naive patient had unknown PD-L1 status owing to a missing laboratory sample and was included in the overall CPI-naive group only (n = 32). ^*c*^Criteria for CPI-naive PD-L1 high cohort from stage 1 to stage 2: if ≥5 responses observed out of 17 assessable patients. Because four responses were observed, the PD-L1 high cohort was not evaluated past stage 1. CPI, checkpoint inhibitor; PCB, prior clinical benefit; NE, not evaluated; NPCB, no prior clinical benefit; PD-L1, programmed cell death-ligand 1; QD, once daily.

A predictive probability design was used in this study for each cohort (Fig. 1).^30^ In CPI-experienced patients, and CPI-naive patients with no or low PD-L1 expression, the target ORR using sitravatinib with nivolumab treatment was assumed to be 30%, and an ORR of 5% was considered uninteresting. Stage 1 of enrollment planned to include nine assessable patients; if at least one patient had an objective response, eight additional assessable patients would be enrolled in stage 2; if at least three objective responses were observed from stages 1 and 2, further investigation was warranted (Fig. 1). For stages 1 and 2, if patients discontinued treatment or withdrew consent before the first on-study disease assessment, they were considered as non-assessable. The type 1 error was 0.0466 and power was 0.9045.

In CPI-naive patients with high PD-L1 expression, the target ORR with sitravatinib and nivolumab treatment was assumed to be 50%, and an ORR of 27% was considered uninteresting. Stage 1 of enrollment was planned to include 17 assessable patients; if at least five patients had an objective response, 27 additional assessable patients would be enrolled in stage 2. If at least 17 objective responses were observed at stage 2, further investigation was warranted (Fig. 1). The type 1 error was 0.0303 and the power was 0.9018. If the threshold for responses achieved in stages 1 and 2 were met, enrollment could be expanded to as many as 125 patients total in the CPI-experienced cohort and 100 patients total in the CPI-naive cohort (Fig. 1). Enrollment of CPI-naive patients beyond stage 2 was stopped owing to the changing treatment landscape—that is, because first-line CPI was becoming standard of care.

All patients who received at least one dose of study treatment were included in the efficacy and safety analyses. The Kaplan-Meier method was used to estimate the median DOR, PFS, OS, and the 1-year survival rate.

## Results

### Patients

As of January 25, 2022, the data cutoff (study start: November 7, 2016), 124 CPI-experienced (89 with PCB from CPI, 35 with NPCB) and 32 CPI-naive patients (20 with no or low PD-L1 expression, 11 with high PD-L1 expression, and one with unknown PD-L1 status) were treated (Supplementary Fig. 1). In CPI-experienced patients with PCB, the median age was 67 years, and 67.4% were former smokers (Table 1). The median number of previous regimens was two (range: 1–10) and 76.4% had received one or two previous lines of therapy. Previous CPI included pembrolizumab (56.2%) and nivolumab (37.1%), whereas 78.7% of patients had also previously received platinum-based chemotherapy. The median number of lines of therapy after platinum-based chemotherapy was one (range: 0–7). The baseline characteristics of each cohort were as expected, with the CPI-experienced and CPI-naive cohorts differing primarily with regard to previous therapy (Table 1).

The patients in the dose escalation cohort were the first six dose-limiting toxicity assessable patients enrolled. These patients were treated with 120 mg and reported no dose-limiting toxicities. On the basis of these data and the long-term tolerability of sitravatinib 150 mg observed in the phase 1/1b 516–001 trial,^22^ sitravatinib 120 mg was evaluated in all subsequent patients; therefore, these initial patients were included in subsequent efficacy and safety analyses and sitravatinib 150 mg was not evaluated (See the Supplementary Appendix for further information).

### Efficacy

The responses observed in the first nine assessable patients (stage 1) and the first 17 assessable patients (stages 1 and 2) are illustrated in Figure 1. On the basis of the responses achieved in stages 1 and 2, enrollment of CPI-experienced patients was expanded to 124 patients, whereas enrollment of CPI-naive patients beyond stage 1 (PD-L1 high subgroup) and beyond stage 2 (PD-L1 no or low subgroup) was stopped at 32 owing to the changing treatment landscape.

**Figure 2.**
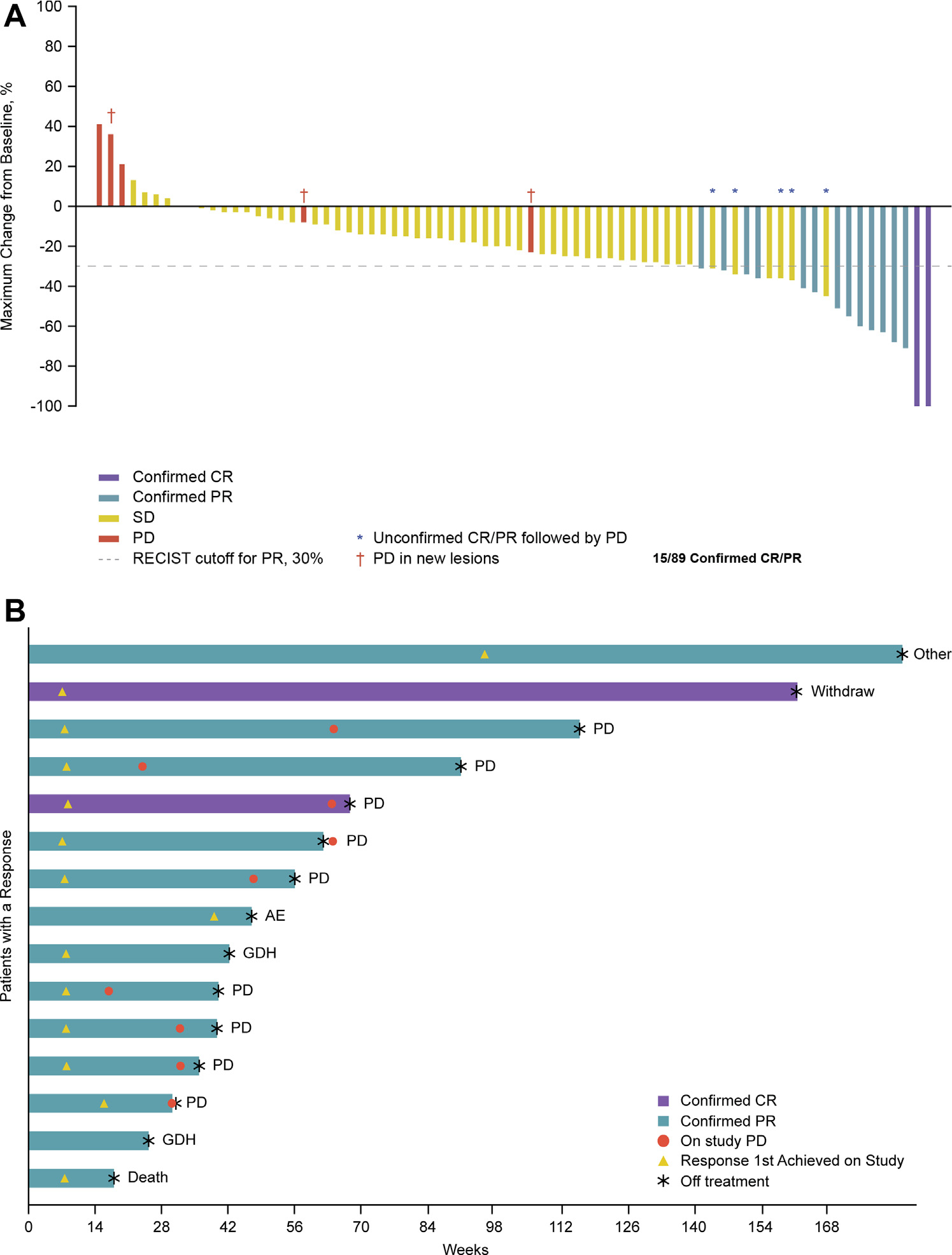
Efficacy of sitravatinib and nivolumab in patients with nonsquamous non-small cell lung cancer previously treated with CPI. (A) Best overall response in patients with PCB from CPI (n = 89). (*B*) Duration of treatment in patients with PCB from CPI who responded to treatment (n = 15). One patient with confirmed PR (indicated with ‘Other’) was enrolled in a rollover study (NCT04887870). AE, adverse event; CPI, checkpoint inhibitor therapy; CR, complete response; GDH, global deterioration of health; PCB, prior clinical benefit; PD, progressive disease; PR, partial response; RECIST v1.1, Response Evaluation Criteria in Solid Tumors version 1.1; SD, stable disease.

**Figure 3.**
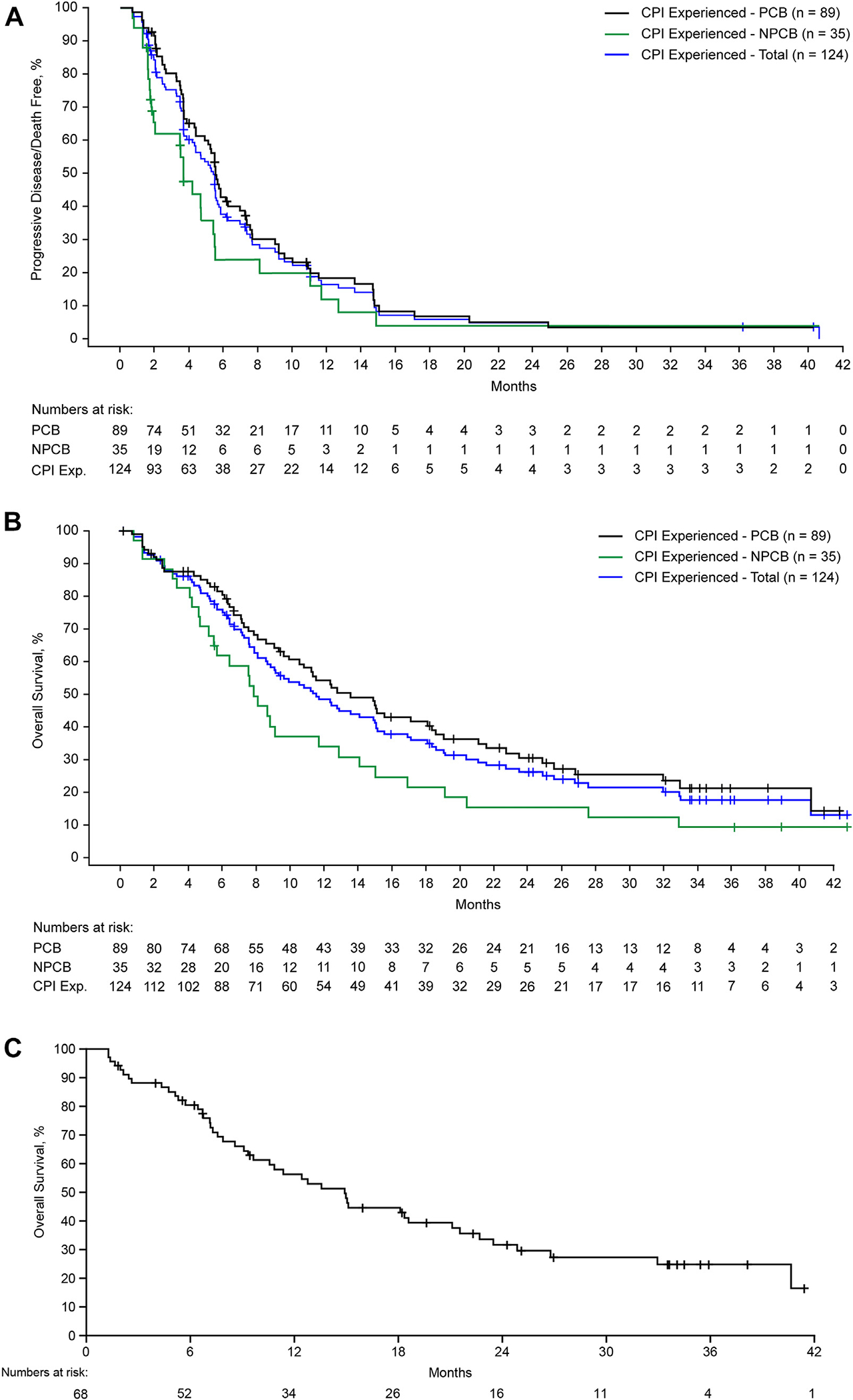
PFS and OS in patients with nonsquamous NSCLC treated with sitravatinib and nivolumab who progressed on or after prior CPI. (A) PFS in CPI-experienced patients with PCB (n = 89), NPCB (n = 35), and total (N = 124). (B) OS in CPI-experienced patients with PCB (n = 89), NPCB (n = 35), and total (N = 124). (C) OS in CPI-experienced patients with PCB from one or two prior lines of therapy (n = 68) (median follow-up = 33.6 mo). Data as of January 25, 2022. Months (x-axis) were calculated from when patients received their first dose. CPI, checkpoint inhibitor therapy; Exp., experienced; NPCB, no prior clinical benefit; OS, overall survival; PCB, prior clinical benefit; PFS, progression-free survival.

The median follow-up for CPI-experienced patients with PCB was 33.5 months. The ORR was 16.9% (15 of 89), including two CRs (2.2%) and 13 PRs (14.6%) (Table 2 and Fig. 2A). Of the 15 patients who achieved a response, 12 had at least one dose reduction, and seven had achieved a PR to their previous CPI. CBR was 78.7% (70 of 89) and the median DOR was 9.2 months (95% confidence interval [CI]: 3.6–13.1) (Table 2 and Fig. 2B). The median PFS was 5.6 months (95% CI: 4.4–7.0), with 6, 9, and 12-month PFS rates of 42.8% (95% CI: 31.6–53.4), 30.1% (95% CI: 20.2–40.7), and 18.2% (95% CI: 10.3–28.0), respectively (Table 2 and Fig. 3A). The median OS was 13.6 months (95% CI: 10.0–18.3), with an OS rate of 54.3% (95% CI: 42.9–64.3) and 30.6% (95% CI, 20.7–41.0) at 12 and 24 months, respectively (Table 2 and Fig. 3B). Notably, in the 68 patients with one or two previous lines of therapy (median follow-up = 33.6 mo), the median OS was 14.9 months (95% CI: 9.3–21.1), with 12- and 24-month OS rates of 56.2% (95% CI: 43.2–67.4) and 31.7% (95% CI: 20.2–43.7), respectively (Fig. 3C).

For CPI-experienced patients with NPCB, the median follow-up was 38.9 months. The ORR was 11.4% (4 of 35; all PRs) (Supplementary Fig. 2); the CBR was 60.0% (21 of 35) and the median DOR was 11.2 months (95% CI: 9.9–not estimable [NE]) (Table 2). The median PFS was 3.7 months (95% CI: 1.8–5.4), with 6, 9, and 12-month PFS rates of 23.8% (95% CI: 10.0–40.9), 19.8% (95% CI: 7.4–36.6), and 11.9% (95% CI: 3.1–27.3), respectively (Table 2 and Fig. 3A). The median OS was 7.9 months (95% CI: 5.2–11.7), with an OS rate of 34.0% (95% CI: 18.7–50.0) and 15.4% (95% CI: 5.7–29.7) at 12 and 24 months, respectively (Table 2 and Fig. 3B).

For CPI-naive patients overall (who progressed on or after platinum-based doublet chemotherapy), median follow-up was 20.4 months and ORR was 25.0% (8 of 32), including one CR (3.1%) and seven PRs (21.9%). For CPI-naive patients with known PD-L1 status, the ORR was 20.0% (four PRs) among 20 patients with no or low PD-L1 expression and 36.4% (one CR, three PRs) among 11 patients with high PD-L1 expression. Overall, the median OS was not reached and the 12- and 24-month OS rates were 68.6% (95% CI: 48.1–82.3) and 60.0% (95% CI: 39.1–75.7), respectively. For CPI-naive patients with known PD-L1 status, the median OS was not reached in either group of patients; for those patients with no/low PD-L1 expression, the 12- and 24-month OS rates were 59.2% (95% CI: 32.7–78.2) and 51.8% (95% CI: 25.8–72.7), respectively; among patients with high PD-L1 expression, the 12- and 24-month OS rates were 90.0% (95% CI: 47.3–98.5) and 78.8% (95% CI: 38.1–94.3), respectively (see Table 2 and Supplementary Fig. 3 for further efficacy results in patients with no or low PD-L1 expression or high PD-L1 expression).

### Safety

The median number of cycles of sitravatinib plus nivolumab was four in all CPI-experienced patients and five in CPI-naive patients. In the overall safety population (N = 156), any-grade treatment-related adverse events (TRAEs) occurred in 146 of 156 patients (93.6%); the most frequent were diarrhea (54.5%), fatigue (46.2%), nausea (39.7%), and decreased appetite (34.6%) (Table 3). Grade 3 and higher TRAEs occurred in 91 of 156 (58.3%) patients; the most common were hypertension (16.7%) and diarrhea (12.8%). Investigator-assessed immune-related adverse events occurred in 68 of 156 patients (43.6%); the most frequent were hypothyroidism (19.2%), pneumonitis (4.5%), and hyperthyroidism, increased blood thyroid stimulating hormone, and rash (each in 3.8%). TRAEs in the CPI-experienced and CPI-naive cohorts were similar to the overall safety population (Table 3). No grade 5 TRAEs occurred in the CPI-experienced cohort; one CPI-naive patient experienced a TRAE (cardiac arrest) leading to death after one dose of nivolumab and four doses of sitravatinib; this patient had a history of radiotherapy to the mediastinum and three previous lines of systemic chemotherapy, including cisplatin, carboplatin, vinorelbine, paclitaxel, and pemetrexed.

Overall, 22 of 156 patients (14.1%) discontinued treatment owing to TRAEs (Table 3); the most common were pneumonitis (3.2%; grade 3), fatigue, and decreased weight (each in 1.9%); discontinuation rates owing to TRAEs were 8.3% for sitravatinib and 9.6% for nivolumab. TRAEs leading to sitravatinib discontinuation included diarrhea, fatigue, and decreased weight (each in 1.3%); TRAEs leading to nivolumab discontinuation included pneumonitis (3.2%), fatigue (1.9%), and decreased weight (1.3%). In addition, TRAEs led to dose reduction/interruption (for sitravatinib defined as missing ≥1 d of study drug) of either study drug in 67 of 156 patients (42.9%); the most common were diarrhea (16.7%) and fatigue (5.8%). The median treatment intensity and dose reductions/interruptions in CPI-experienced and CPI-naive patients are presented in Supplementary Table 1.

### Biomarker Analyses

Exploratory analyses evaluated the mechanism of action of sitravatinib plus nivolumab. Patients with higher (≥50%) baseline PD-L1 staining tended to have a higher likelihood of deriving clinical benefit (*p* = 0.0552) (Supplementary Fig. 4A). In retrospective analyses, selected cancer-associated mutations and alterations that are putative targets of sitravatinib in vitro (e.g., RET, MET, CBL, AXL, KDR, and NTRK) were monitored from circulating tumor DNA in baseline plasma samples (n = 49). Most patients with clinical benefit did not have alterations in sitravatinib-targeted tyrosine kinases and only four patients with clinical benefit had these tyrosine kinase alterations (one MET mutation, two CBL mutations,^31^ one RET fusion) (Supplementary Fig. 4B), suggesting that clinical benefit may be derived from other mechanisms such as effects on the TME. Among the four patients who had STK11 mutations, two had the clinical benefit (Supplementary Fig. 4B). Tumor mutational burden did not correlate with clinical benefit. Flow cytometry was performed on blood samples obtained during pretreatment and at cycle 1 (n = 95), day 15 postcombination treatment (Supplementary Fig. 4C). MDSCs and Tregs were decreased, whereas CD8+ T cells and activated CD8+ T cells (CD45RA+, CD62L−) were increased in postcombination treated blood samples.

## Discussion

Acquired resistance to CPI is typically defined by a period of initial clinical benefit from CPI therapy, followed by clinical or radiological progression of the disease. However, given that tumor resistance patterns to CPI therapy are often heterogenous, defining resistance has proven challenging, and there is a lack of consensus in the field.^32–34^

In addition, treatment options for patients who develop disease progression while receiving initial CPI and chemotherapy are limited. Because TAM receptors and VEGFR2 contribute to an immunosuppressive TME (a suggested mechanism of CPI resistance),^13^ targeting these receptors may have implications on cancer immunotherapy.^19,21^ Sitravatinib, a TKI targeting TAM receptors and VEGFR2, in combination with nivolumab, a PD-1 inhibitor, may, therefore, overcome CPI resistance and an immunosuppressive TME.^17^ In this phase 2 study, although the primary end point of ORR was not met, sitravatinib with nivolumab exhibited durable responses and an encouraging median OS of 13.6 months in patients with NSQ NSCLC with PCB from CPI. In contrast to unsuccessful historical attempts to combine CPI with TKIs,^35,36^ there were no unexpected safety signals in this study, and the safety profile was manageable with the most frequent TRAEs (e.g., fatigue, nausea, decreased appetite) and TRAEs leading to treatment discontinuation (e.g., pneumonitis) also being reported previously with nivolumab monotherapy.^1^ In addition, dose modification rates were similar to those seen in this class of TKIs.^37^

For patients receiving second-line regimens containing docetaxel with or without previous CPI, ORR is typically 9% to 27%,^38^ as revealed in the phase 3 REVEL^39^ and CANOPY-2 studies.^40^ A retrospective, observational study in the United States, using the Flatiron Health database, found that patients with subsequent treatment after progression on CPI and chemotherapy had a median OS of 7.3 months; the most common treatments were docetaxel plus ramucirumab (median OS = 6.5 mo) and docetaxel monotherapy (median OS = 6.7 mo).^41^ Moreover, the recent phase 2 Lung-MAP S1800A study found that patients receiving standard-of-care chemotherapy (typically docetaxel and ramucirumab) after progression on CPI and chemotherapy had a median OS of 11.6 months.^42^ In our study, patients with PCB from CPI who were treated with sitravatinib plus nivolumab had a median OS of 13.6 months, with a 12- and 24-month survival rate of 54.3% and 30.6%, respectively. In patients receiving sitravatinib and nivolumab as second or third-line treatment with a previous clinical benefit, the median OS was 14.9 months, and the 12-month survival rate was 56.2%.

The biomarker analyses conducted as part of this study support the hypothesis that sitravatinib dampens the immunosuppressive TME and may synergize with CPI therapy.^17^ The combination regimen led to increased levels of peripheral CD8+ and activated CD8+ T cells, while decreasing MDSCs and Treg cells, indicating that sitravatinib inhibits key immune suppressive cell populations and redirects the immune system toward an antitumor response. Most patients who derived the benefit did not have alterations in putative tyrosine kinase targets of sitravatinib, meaning that this benefit may have been achieved through a nononcogenic driver mechanism and potentially through inhibition of targets in the TME. STK11 mutation is emerging as a biomarker associated with CPI resistance in NSCLC.^43,44^ Interestingly, we observed that two out of four patients with STK11 mutations had a clinical benefit in our study, which might suggest an anecdotal clinical activity signal of the trial treatment in this population.

Increased baseline PD-L1 tended to be associated with clinical benefit, suggesting that sitravatinib resensitizes at least a subset of tumors to CPI and is in line with previous data suggesting that CPIs have increased activity in PD-L1-high cancers.^1,43,45^ Given the complexity of the TME, further research, including randomized trials, is needed to accurately select biomarkers and patients for immunotherapy and immunotherapy-based regimens.^46^

A clear limitation of this study is the single-arm design, which precluded patient randomization to the current standard of care. The lack of a universal definition of CPI-acquired resistance and PCB meant this was defined subjectively but on the basis of clinical judgment. Tissue biopsy collection was not a requirement, limiting our ability to perform robust tissue translational analyses to further evaluate data from those patients who derived a clinical benefit. The analyses of PD-L1 levels should be taken with caution, as these were performed on baseline samples before immunotherapy and conducted using different methods. In addition, disease progression before enrollment in CPI-experienced patients was investigator-assessed and not confirmed centrally, potentially leading to biases in the patient population.

The promising OS in this study, particularly in patients receiving sitravatinib and nivolumab as a second- or third-line treatment, has provided the basis for the ongoing phase 3 SAPPHIRE trial (NCT03906071)^47^; this study is evaluating sitravatinib plus nivolumab versus docetaxel in patients with advanced NSQ NSCLC who received a clinical benefit from, and subsequently progressed on, previous CPI and chemotherapy as first-or second-line treatments. Another phase 3 study (NCT04921358)^26^ is evaluating sitravatinib plus tislelizumab (a PD-1 inhibitor) in patients with locally advanced/metastatic NSCLC who experienced disease progression after CPI and chemotherapy. The phase 3 CONTACT-01 study (NCT04471428) evaluating cabozantinib plus atezolizumab did not meet its primary end point of OS at the final analysis,^48^ but further studies investigating other TKIs against TAM receptors are ongoing, such as bemcentinib (an AXL TKI) in combination with pembrolizumab in phase 2 BGBC008 trial (NCT03184571)^49^ and INCB081776 (an AXL/MER TKI) in a phase 1 trial (NCT03522142).^50^

In conclusion, sitravatinib with nivolumab exhibited favorable DOR and encouraging OS along with a manageable safety profile in patients with NSQ NSCLC who progressed on or after previous CPI.

## Supplementary Material

1

## Figures and Tables

**Table 1.** Demographics and Baseline Characteristics of CPI-Experienced and CPI-Naive Patients With NSQ NSCLC Who Progressed on or After Previous CPI or Chemotherapy

	CPI-Experienced			CPI-Naive		
Characteristics	PCB (n = 89)	NPCB (n = 35)	Overall (N = 124)	PD-L1 no or low (n = 20)	PD-L1 high (n = 11)	Overall (N = 32)^a^

Median age, y (range)	67.0 (37–87)	65.0 (37–84)	66.0 (37–87)	66.0 (48–89)	67.0 (30–79)	67.5 (30–89)
Sex, n (%)						
Male	40 (44.9)	18 (51.4)	58 (46.8)	7 (35.0)	5 (45.5)	12 (37.5)
Female	49 (55.1)	17 (48.6)	66 (53.2)	13 (65.0)	6 (54.5)	20 (62.5)
Race, n (%)						
White	78 (87.6)	29 (82.9)	107 (86.3)	16 (80.0)	9 (81.8)	26 (81.3)
Black or African American	6 (6.7)	2 (5.7)	8 (6.5)	3 (15.0)	0	3 (9.4)
Asian	3 (3.4)	0	3 (2.4)	1 (5.0)	0	1 (3.1)
Other	2 (2.2)	4 (11.5)	6 (4.8)	0	2 (18.2)	2 (6.2)
ECOG PS, n (%)						
0	22 (24.7)	9 (25.7)	31 (25.0)	4 (20.0)	4 (36.4)	8 (25.0)
1	60 (67.4)	26 (74.3)	86 (69.4)	16 (80.0)	7 (63.6)	24 (75.0)
2	7 (7.9)	0	7 (5.6)	0	0	0
Smoking status, n (%)						
Never smoker	15 (16.9)	8 (22.9)	23 (18.5)	4 (20.0)	2 (18.2)	6 (18.8)
Current smoker	14 (15.7)	5 (14.3)	19 (15.3)	3 (15.0)	1 (9.1)	4 (12.5)
Former smoker	60 (67.4)	22 (62.9)	82 (66.1)	13 (65.0)	8 (72.7)	22 (68.8)
Current stage, n (%)						
Locally advanced	5 (5.6)	5 (14.3)	10 (8.1)	4 (20.0)	0	4 (12.5)
Metastatic	84 (94.4)	30 (85.7)	114 (91.9)	16 (80.0)	11 (100)	28 (87.5)
Median number of prior regimens, n (range)	2.0 (1–10)	2.0 (1–10)	2.0 (1–10)	1.0 (0–3)	1.0 (0–3)	1.0 (0–3)
Number of prior regimens, n (%)						
0	0	0	0	1 (5.0)	1 (9.1)	2 (6.3)^b^
1	37 (41.6)	12 (34.3)	49 (39.5)	12 (60.0)	7 (63.6)	19 (59.4)
2	31 (34.8)	15 (42.9)	46 (37.1)	5 (25.0)	1 (9.1)	6 (18.8)
≥3	21 (23.6)	8 (22.9)	29 (23.4)	2 (10.0)	2 (18.2)	5 (15.6)
Prior platinum-based chemotherapy, n (%)	70 (78.7)	31 (88.6)	101 (81.5)	19 (95.0)	10 (90.9)	30 (93.8)
Cisplatin	12 (13.5)	5 (14.3)	17 (13.7)	2 (10.0)	2 (18.2)	5 (15.6)
Carboplatin	56 (62.9)	25 (71.4)	81 (65.3)	17 (85.0)	8 (72.7)	25 (78.1)
Other	2 (2.2)^c^	1 (2.9)^d^	3 (2.4)	0	0	0
Median number of lines of therapy post platinum-based chemotherapy, n (range)	1.0 (0–7)	1.0 (0–9)	1.0 (0–9)	NA	NA	0 (0–1)
Number of lines of therapy post platinum-based chemotherapy, n (%)						
0	41 (46.1)	15 (42.9)	56 (45.2)	NA	NA	27 (84.4)
1	31 (34.8)	15 (42.9)	46 (37.1)	NA	NA	5 (15.6)
2	11 (12.4)	2 (5.7)	13 (10.5)	0	0	0
≥3	6 (6.7)	3 (8.6)	9 (7.3)	0	0	0
Prior PD-1/L1 CPI, n (%)	89 (100)	35 (100)	124 (100)	–	–	–
Nivolumab	33 (37.1)	13 (37.1)	46 (37.1)	–	–	–
Pembrolizumab	50 (56.2)	19 (54.3)	69 (55.6)	–	–	–
Durvalumab	1 (1.1)	0	1 (0.8)	–	–	–
Atezolizumab	5 (5.6)	3 (8.6)	8 (6.5)	–	–	–
Best response^e^ to prior CPI, n (%)						
Complete response	2 (2.2)	0	2(1.6)	–	–	–
Partial response	36 (40.4)	0	36 (29.0)	–	–	–
Stable disease	51 (57.3)	0	51 (41.1)	–	–	–
Progressive disease	0	35 (100)	35 (28.2)	–	–	–

a One CPI-naive patient had unknown PD-L1 status owing to a missing laboratory sample and was included in the overall CPI-naive group only.

b Two patients who received no previous treatments were deviations from the protocol.

c Other chemotherapies were cisplatin in combination with carboplatin.

d Other chemotherapy was pemetrexed.

e Based on investigator assessment.

CPI, checkpoint inhibitor therapy; ECOG PS, Eastern Cooperative Oncology Group performance status; NA, not available; NPCB, no prior clinical benefit; NSQ, nonsquamous; PCB, prior clinical benefit; PD-1, programmed cell death protein-1; PD-L1, programmed death-ligand 1.

**Table 2.** Clinical Efficacy of Sitravatinib With Nivolumab in CPI-Experienced and CPI-Naive Patients With NSCLC Who Progressed on or After Previous CPI or Chemotherapy

	CPI-Experienced			CPI-Naive		
Efficacy Outcome	PCB (n = 89)	NPCB (n = 35)	Overall (N = 124)	PD-L1 no/low (n = 20)	PD-L1 high (n = 11)	Overall (N = 32)^a^

Median follow-up, mo	33.5	38.9	34.1	17.5	30.4	20.4
ORR						
n (%)	15 (16.9)	4 (11.4)	19 (15.3)	4 (20.0)	4 (36.4)	8 (25.0)
95% CI	9.8–26.3	3.2–26.7	9.5–22.9	5.7–43.7	10.9–69.2	11.5–43.4
BOR, n (%)^b^						
CR	2 (2.2)	0	2 (1.6)	0	1 (9.1)	1 (3.1)
PR	13 (14.6)	4 (11.4)	17 (13.7)	4 (20.0)	3 (27.3)	7 (21.9)
SD	55 (61.8)	17 (48.6)	72 (58.1)	8 (40.0)	7 (63.6)	15 (46.9)
PD	5 (5.6)	9 (25.7)	14 (11.3)	5 (25.0)	0	5 (15.6)
NE	14 (15.7)^c^	5 (14.3)^d^	19 (15.3)	3 (15.0)	0	4 (12.5)^e^
CBR						
n (%)	70 (78.7)	21 (60.0)	91 (73.4)	12 (60.0)	11 (100.0)	23 (71.9)
95% CI	68.7–86.6	42.1–76.1	64.7–80.9	36.1–80.9	71.5–100.0	53.3–86.3
DOR^f^						
Median, mo (95% CI)	9.2 (3.6–13.1)	11.2 (9.9-NE)	11.0 (3.7–13.1)	7.6 (3.7-NE)	NR (5.8-NE)	11.1 (3.7-NE)
PFS						
Median, mo (95% CI)	5.6 (4.4–7.0)	3.7 (1.8–5.4)	5.4 (4.2–5.7)	6.7 (1.9–10.1)	13.0 (5.4-NE)	7.1 (4.0–13.1)
6-mo KM estimate, % (95% CI)	42.8 (31.6–53.4)	23.8 (10.0–40.9)	37.7 (28.5–46.9)	51.8 (26.2–72.4)	87.5 (38.7–98.1)	62.3 (41.0–77.8)
9-mo KM estimate, % (95% CI)	30.1 (20.2–40.7)	19.8 (7.4–36.6)	27.4 (19.1–36.3)	38.8 (16.3–61.1)	62.5 (22.9–86.1)	45.3 (25.4–63.3)
12-mo KM estimate, % (95% CI)	18.2 (10.3–28.0)	11.9 (3.1–27.3)	16.4 (9.8–24.5)	23.3 (6.2–46.7)	50.0 (15.2–77.5)	31.7 (14.5–50.4)
OS						
Median, mo (95% CI)	13.6 (10.0–18.3)	7.9 (5.2–11.7)	11.5 (8.7–15.0)	NR (6.7-NE)	NR (7.8-NE)	NR (9.8-NE)
12-mo KM estimate, % (95% CI)	54.3 (42.9–64.3)	34.0 (18.7–50.0)	48.5 (39.1–57.2)	59.2 (32.7–78.2)	90.0 (47.3–98.5)	68.6 (48.1–82.3)
18-mo KM estimate, % (95% CI)	41.6 (30.8–52.0)	21.6 (9.6–36.8)	35.9 (27.2–44.7)	51.8 (25.8–72.7)	78.8 (38.1–94.3)	60.0 (39.1–75.7)
24-mo KM estimate, % (95% CI)	30.6 (20.7–41.0)	15.4 (5.7–29.7)	26.2 (18.4–34.7)	51.8 (25.8–72.7)	78.8 (38.1–94.3)	60.0 (39.1–75.7)

a One CPI-naive patient had unknown PD-L1 status owing to a missing laboratory sample and was included in the overall CPI-naive group only.

b A confirmed BOR of CR or PR requires a confirmatory assessment ≥4 weeks (≥28 d) because of the first CR or PR response. A CR or PR response without confirmation is summarized as SD if the assessment is >42 days after the date of the first dose and is summarized as NE if it is <42 days from the date of the first dose. For a BOR of SD, an SD assessment must be ≥42 days from the date of the first dose, otherwise, it will be summarized as NE.

c A total of 13 patients had no post-baseline scans (including one patient with no measurable disease at baseline) owing to discontinuing study treatment before undergoing the first on-study disease assessment. Reasons for discontinuing study treatment included adverse events (n = 4), withdrawal by patient (n = 3), death (n = 3), global deterioration of health (n = 2), and investigator decision (n = 1). One patient had NE post-baseline scans.

d Three patients had no measurable disease at baseline and two had no post-baseline scans.

e Two patients had baseline-only scans, one had no baseline/post-baseline scans, and one had NE post-baseline scans.

f DOR was analyzed in those patients who achieved an objective response (CR or PR).

BOR, best overall response; CBR, clinical benefit rate; CI, confidence interval; CPI, checkpoint inhibitor therapy; CR, complete response; DOR, duration of response; KM, Kaplan-Meier; NE, not assessable; NPCB, no prior clinical benefit; NR, not reached; ORR, overall response rate; OS, overall survival; PCB, prior clinical benefit; PFS, progression-free survival; PD, progressive disease; PD-L1, programmed death-ligand 1; PR, partial response; SD, stable disease.

**Table 3.** Incidence of TRAEs in the Overall Safety Population

	CPI-Expereinced (N = 124)							
	PCB (n = 89)		NPCB (n = 35)		CPI-Naive (n = 32)		Overall (N = 156)	
TRAEs, n (%)	Any Grade	Grade 3/4	Any Grade	Grade 3/4	Any Grade	Grade 3/4^a^	Any Grade	Grade 3/4^a^

Any TRAEs	80 (89.9)	52 (58.4)	34 (97.1)	21 (60.0)	32 (100)	18 (56.3)	146 (93.6)	91 (58.3)
Most Frequent TRAEs (≥15% Any Grade in Overall Population), n (%)	Any Grade	Grade 3/4	Any Grade	Grade 3/4	Any Grade	Grade 3/4	Any Grade	Grade 3/4
Diarrhea	53 (59.6)	14 (15.7)	13 (37.1)	5 (14.3)	19 (59.4)	1 (3.1)	85 (54.5)	20 (12.8)^b^
Fatigue	40 (44.9)	4 (4.5)	17 (48.6)	2 (5.7)	15 (46.9)	3 (9.4)	72 (46.2)	9 (5.8)
Nausea	36 (40.4)	1 (1.1)	12 (34.3)	1 (2.9)	14 (43.8)	1 (3.1)	62 (39.7)	3 (1.9)
Decreased appetite	30 (33.7)	0(0)	9 (25.7)	1 (2.9)	15 (46.9)	0(0)	54 (34.6)	1 (0.6)
Hypertension	29 (32.6)	16 (18.0)	8 (22.9)	4 (11.4)	9 (28.1)	6 (18.8)	46 (29.5)	26 (16.7)^b^
Weight decreased	27 (30.3)	5 (5.6)	12 (34.3)	3 (8.6)	5 (15.6)	1 (3.1)	44 (28.2)	9 (5.8)
Vomiting	26 (29.2)	0(0)	8 (22.9)	2 (5.7)	7 (21.9)	1 (3.1)	41 (26.3)	3 (1.9)
Hypothyroidism	18 (20.2)	0(0)	10 (28.6)	0(0)	12 (37.5)	1 (3.1)	40 (25.6)	1 (0.6)
Dysphonia	20 (22.5)	0(0)	5 (14.3)	0(0)	6 (18.8)	0(0)	31 (19.9)	0(0)
PPE syndrome	12 (13.5)	2 (2.2)	7 (20.0)	1 (2.9)	8 (25.0)	1 (3.1)	27 (17.3)	4 (2.6)
AST increase	14 (15.7)	0(0)	7 (20.0)	2 (5.7)	5 (15.6)	0(0)	26 (16.7)	2(1.3)
Stomatitis	13 (14.6)	2 (2.2)	7 (20.0)	0(0)	4 (12.5)	0(0)	24 (15.4)	2(1.3)

TRAEs, n (%)	Any grade		Any grade		Any grade		Any grade	

TRAEs leading to treatment discontinuation	12 (13.5)		6 (17.1)		4 (12.5)		22 (14.1)	
TRAEs leading to sitravatinib discontinuation	9 (10.1)		3 (8.6)		1 (3.1)		13 (8.3)	
TRAEs leading to nivolumab discontinuation	7 (7.9)		5 (14.3)		3 (9.4)		15 (9.6)	

a One grade 5 TRAE (cardiac arrest) occurred in a CPI-naive patient.

b Median time to onset of diarrhea or hypertension from day 1 of treatment was 50.0 days for patients with PCB and 42.5 days for patients with NPCB.

AST, aspartate aminotransferase; CPI, checkpoint inhibitor therapy; NPCB, no prior clinical benefit; PCB, prior clinical benefit; PPE, palmar-plantar eryth- rodysesthesia; TRAEs, treatment-related adverse events.

## Data Availability

At Mirati Therapeutics we are committed to patient care, advancing scientific understanding, and enabling the scientific community to learn from and build on the research we have undertaken. To that end, we will honor legitimate requests for our clinical trial data from qualified researchers and investigators for conducting methodologically sound research. We will share clinical trial data, clinical study reports, study protocols, and statistical analysis plans from clinical trials for which results have been posted on clinicaltrials.gov for products and indications approved by regulators in the U.S. and/or EU. Sharing is subject to the protection of patient privacy and respect for the patient’s informed consent. In general, data will be made available for specific requests approximately 24 months after clinical trial completion from our in-scope interventional trials. For additional information on proposals with regard to data-sharing collaborations with Mirati, please e-mail us at medinfo@mirati.com.
