# Supplementary material for: MRTX-500 Phase 2 Trial: Sitravatinib With Nivolumab in Patients With Nonsquamous NSCLC Progressing On or After Checkpoint Inhibitor Therapy or Chemotherapy
Source: J Thorac Oncol. Author manuscript; Available in PMC 2024 Jul 1. (PMC10330304; doi:10.1016/j.jtho.2023.02.016)
Supplement: 1 [file NIHMS1895208-supplement-1.docx]

**Supplementary Appendix**

**Methods**

*Study design*

A dose-limiting toxicity was defined as any grade 4 non-hematological toxicity, grade 3 non-hematological toxicity, or grade 3/4 hematological toxicity that does not recover to grade ≤2, grade 2 pneumonitis or colitis that does not resolve to grade ≤1, febrile neutropenia or neutropenia associated with systemic infection, or toxicity that requires suspension of treatment for >2 weeks. The maximum tolerated dose was defined as the highest sitravatinib dose administered in the combination regimen associated with the decision to ‘stay with the current dose’ using the experience of at least six patients during the first 28-day treatment cycle.

The study dose selected was the highest dose associated with: sufficient safety/tolerability to anticipate that patients will typically be able to receive treatment with ≥75% of the intended dose intensity of sitravatinib and 100% dose intensity of nivolumab; and no observed grade ≥3 or serious immune-related adverse events causally related to the combination regimen.

*Study objectives and assessments*

Survival status and subsequent therapies were collected during long-term follow-up every 2 months from the end of treatment visit until death or loss to follow-up.

Screening and baseline tumor assessments were conducted up to 4 weeks before first study treatment. On-study tumor assessments were performed every 8 weeks until ~1 year and then every 16 weeks. Assessments were performed until investigator-assessed objective disease progression or until subsequent anticancer therapy was started. Adverse events (AEs) were graded using the National Cancer Institute Common Terminology Criteria for Adverse Events version 4.03. Patients were monitored for AEs for ≥28 days after the last dose of study treatment.

The reporting period for non-serious AEs started from the first dose of study treatment until at least 28 days after last administration of study treatment and/or until recovery from all acute toxicities associated with the drug administration to a chronic condition, whichever occurred later. If a patient initiated a subsequent anticancer therapy, the AE reporting period ended at the time the new treatment started.

*Definitions of key efficacy endpoints*

Clinical benefit was defined as patients with complete response (CR), partial response (PR), and stable disease (SD) for ≥12 weeks. No prior clinical benefit was defined as radiographic progression of disease (PD) ≤12 weeks after initiation of treatment. Duration of response was defined as the time from date of the first documentation of objective tumor response (CR or PR) to the first documentation of objective PD or to death due to any cause in the absence of documented PD. Progression-free survival was defined as the time from date of first study treatment to first PD or death due to any cause in the absence of documented PD. Overall survival was defined as the time from date of first study treatment to death due to any cause.

*Biomarker Analyses*

All analyses were performed in R (version 4.0.2)^1^

Programmed death-1-ligand (PD-L1) protein expression levels were obtained from 76 patients among all cohorts. PD-L1 expression was measured at a central laboratory using formalin-fixed paraffin-embedded tumor sections by immunohistochemistry using the Dako 28-8 pharmDx, which generated a PD-L1 score of either <1%, 1–49%, or ≥50%. Prior PD-L1 results using a local test were also allowed for CPI-experienced patients. A Pearson’s chi-squared test with two degrees of freedom was used to determine whether there were significant differences in the frequencies of PD-L1 staining percentages in patients with clinical benefit or no clinical benefit.

Circulating tumor DNA from 49 patients at the screening time point were analyzed in a retrospective analysis using the OMNI 500 gene panel at Guardant Health. Tumor mutational burden (TMB) was analyzed as a continuous variable; the total number of non-silent mutations in 500 genes in each sample, without any normalization to gene length, was used as an approximation of TMB. Mutations in DNMT3A, GNAS, JAK2, TET2, ASXL1, PPM1D, SFB1 were not included in the calculation of total number of mutations per sample. The Kruskal-Wallis rank sum test was used to compare TMB in patients with clinical benefit versus no clinical benefit.

Flow cytometry samples collected from 95 patients per the protocol were analyzed at a central laboratory and data that passed quality check was normalized using the Box-Cox power transformation (forecast v8.15^2^, car v3.0-10^3^) and scaled. Mixed linear models (lme4, v1.1-27^4^) were used to compare cycle 1 day 15 to cycle 1 day 1 with patient ID as a covariate. Response was defined as confirmed CR or confirmed PR. No response was defined as PD and SD.

**Supplementary Table 1.** Sitravatinib treatment intensity, dose reduction, and dose interruption rates in patients with NSQ NSCLC who progressed on or after prior CPI or chemotherapy.

|  | **CPI-experienced (n = 124)** | | **CPI-naïve (n = 32)** |
| --- | --- | --- | --- |
|  | **PCB (n = 89)** | **NPCB (n = 35)** |  |
| Median sitravatinib treatment duration, weeks | 18.6 | 14.1 | 18.7 |
| Relative dose intensity of sitravatinib, median % | 76.4 | 99.1 | 79.1 |
| ≥1 dose reduction of sitravatinib,  n (%) | **51 (57.3)** | **17 (48.6)** | **15 (46.9)** |
| Any AEs | 49 (55.1) | 16 (45.7) | 15 (46.9) |
| Other | 2 (2.2) | 1 (2.9) | 0 (0) |
| Dose reduction of sitravatinib due to AEs, n (%) |  |  |  |
| 80 mg | 26 (29.2) | 8 (22.9) | 8 (25.0) |
| 60 mg | 18 (20.2) | 5 (14.3) | 6 (18.8) |
| 40 mg | 5 (5.6) | 3 (8.6) | 1 (3.1) |
| ≥1 dose interruption of sitravatinib,  n (%) | **86 (96.6)** | **33 (94.3)** | **30 (93.8)** |
| Any AEs | 70 (78.7) | 29 (82.9) | 28 (87.5) |
| Patient non-compliance | 3 (3.4) | 2 (5.7) | 2 (6.3) |
| Missed dose | 15 (16.9) | 7 (20.0) | 9 (28.1) |
| Other | 22 (24.7) | 10 (28.6) | 6 (18.8) |

AEs, adverse events; CPI, checkpoint inhibitor therapy; NPCB, no prior clinical benefit; NSQ NSCLC, non-squamous non-small cell lung cancer; PCB, prior clinical benefit.

**Supplementary Figure 1.** Patient enrollment and disposition.


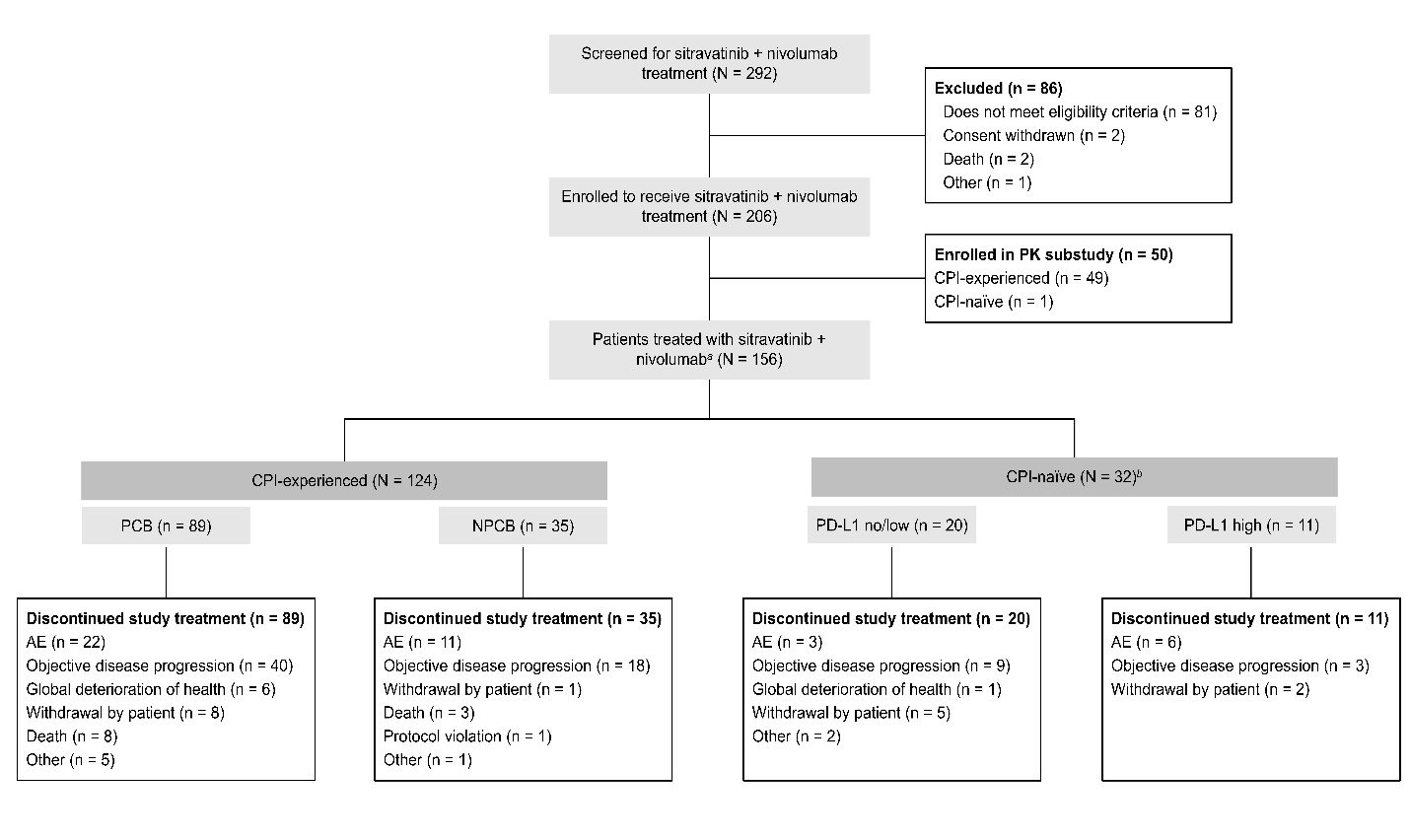


*^a^*All treated patients qualified for the safety population and the efficacy analysis population; *^b^*One CPI-naïve patient had unknown PD-L1 status due to a missing lab sample and was included in the overall CPI-naïve group only.
AE, adverse event; CPI, checkpoint inhibitor therapy; NPCB, no prior clinical benefit; PCB, prior clinical benefit; PD-L1, programmed cell death ligand 1; PK, pharmacokinetic

**Supplementary Figure 2.** Best overall response in patients with NSQ NSCLC treated with sitravatinib and nivolumab with NPCB from CPI (n = 35).

**
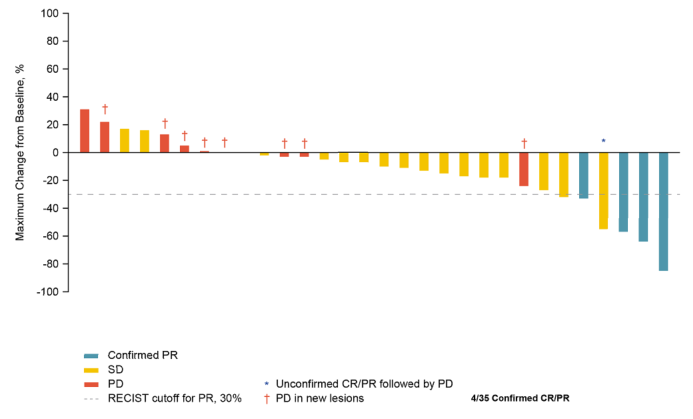
**

CPI, checkpoint inhibitor therapy; CR, complete response; NPCB, no prior clinical benefit; NSQ NSCLC, non-squamous non-small cell lung cancer; PD, progressive disease; PR, partial response; RECIST, Response Evaluation Criteria in Solid Tumors; SD, stable disease.

**Supplementary Figure 3.**  PFS and OS in patients with NSQ NSCLC treated with sitravatinib and nivolumab who progressed on or after prior chemotherapy.

(*A*) PFS in CPI-naïve patients with no/low PD-L1 expression (n = 20), high PD-L1 expression (n = 11), and total (n = 32)^a^.


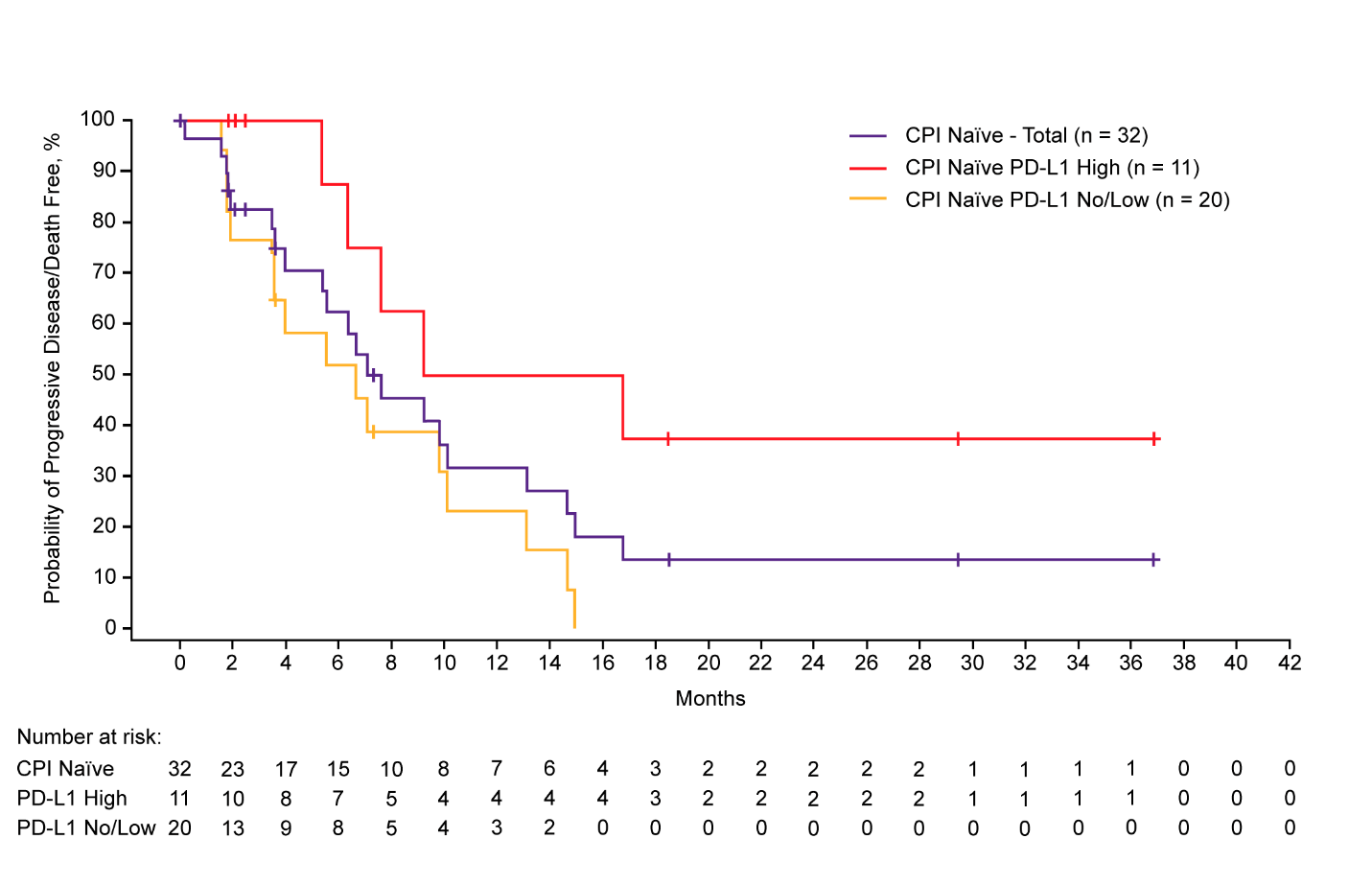


(*B*) OS in CPI-naïve patients with no/low PD-L1 expression (n = 20), high PD-L1 expression (n = 11), and total (n = 32)^a^.


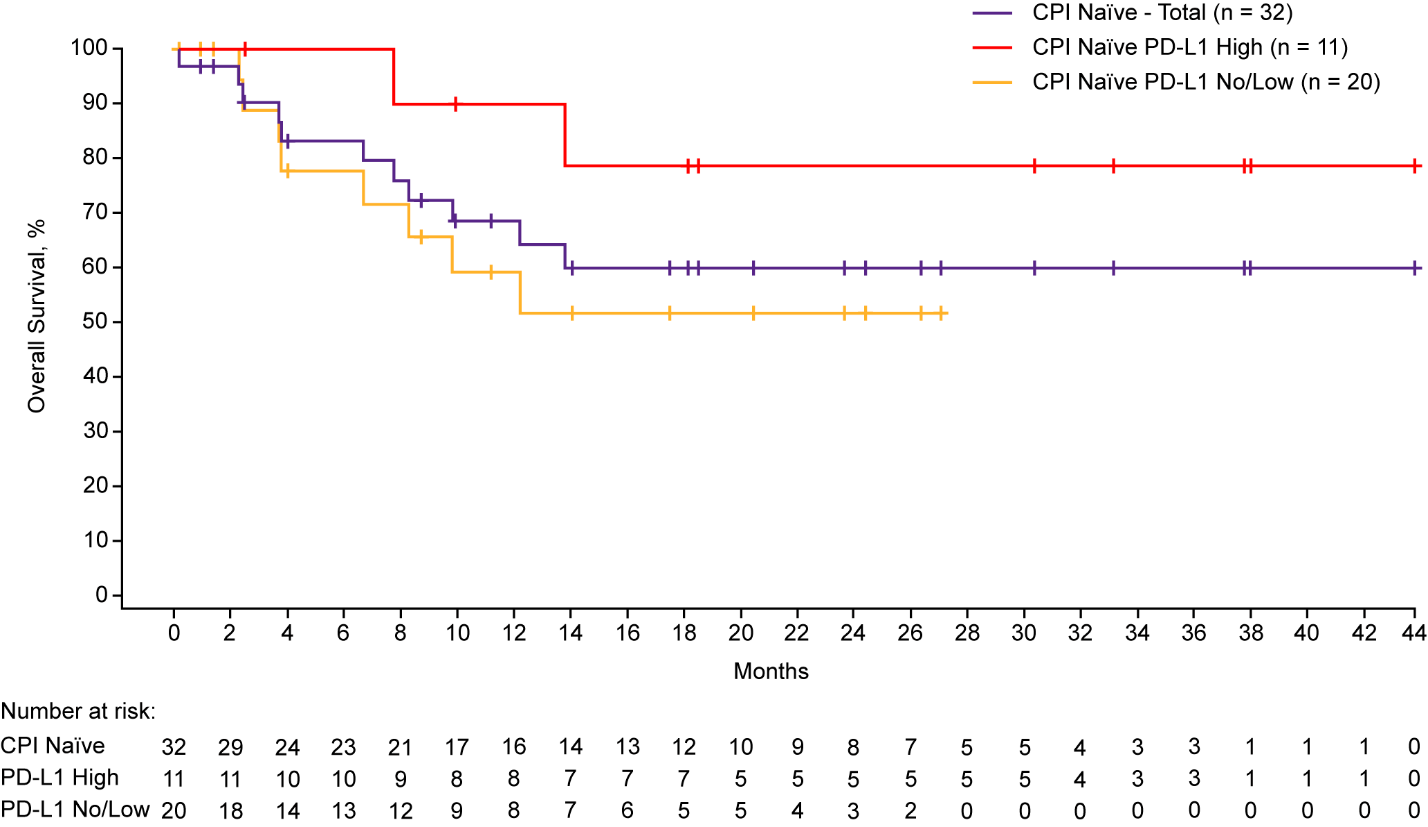


^a^One CPI-naïve patient had unknown PD-L1 status due to a missing lab sample and was included in the overall CPI-naïve group only.

CPI, checkpoint inhibitor; NSQ NSCLC, non-squamous non-small cell lung cancer; OS, overall survival; PD-L1, programmed death-ligand 1; PFS, progression-free survival.

**Supplementary Figure 4.** PD-L1, TMB, and flow cytometry pharmacodynamic data.


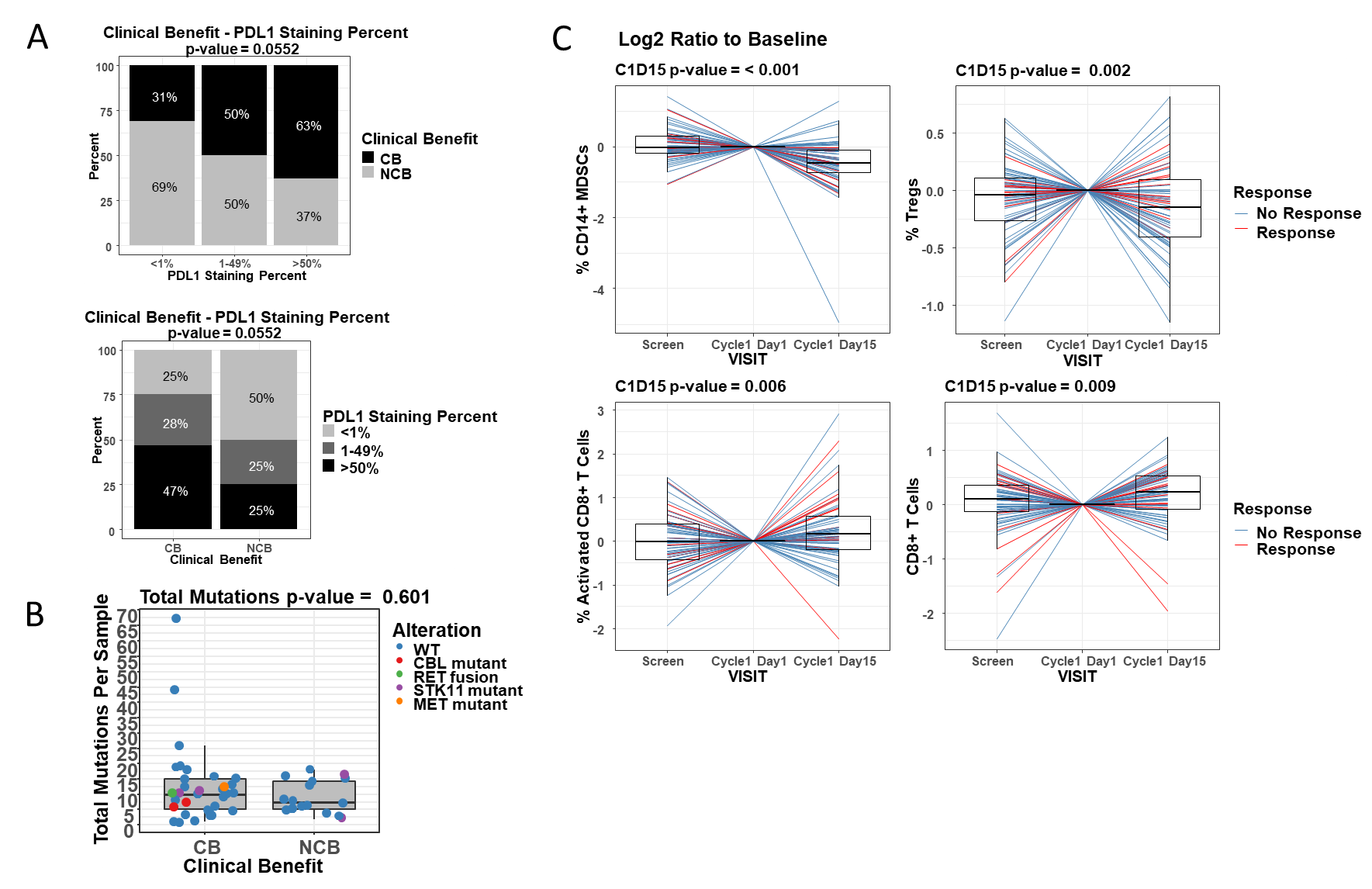


(*A*) PD-L1 staining from 76 patients. (*B*) ctDNA analysis from 49 patients retrospectively analysed. (*C*) Normalized and scaled flow cytometry data from all available patient data normalized to C1D1. *Note*: no appreciable change in the average value was observed between the screen and C1D1 samples (the two pre-treatment samples), however significant changes in the average value between C1D1 and C1D15 were observed for these cell types. Upper left panel – normalized and scaled % of mononuclear cells that are CD14+, HLA-DR low, CD11b+, CD33+, Lin-; upper right panel – normalized and scaled % of cells that are CD127 low, CD25+, CD3+, CD4+, CD45+; lower left panel – normalized and scaled % of lymphocytes that are CD3+, CD4-, CD8+, CD45RA+, CD62L-; lower right panel – normalized and scaled absolute number of lymphocytes that are CD8+, CD3+, CD45+.

C, cycle; CB, clinical benefit (complete response, partial response, and stable disease); CD, cluster of differentiation; ctDNA, circulating tumor DNA; D, day; HLA-DR, human leukocyte antigen DR; Log2, binary logarithm; MDSCs, myeloid-derived suppressor cells; NCB, no clinical benefit; PD-L1, programmed death-ligand 1; TMB, tumor mutational burden; Tregs, regulatory T cells; WT, wild-type.

**REFERENCES**

1. Team RC: R: A language and environment for statistical computing. <https://www.R-project.org/>

2. Hyndman RJ, Khandakar Y: Automatic time series forecasting: the forecast package for R. J Stat Softw 27:1-22, 2008

3. Fox J, Weisberg S: An {R} Companion to Applied Regression (ed 3), Sage, 2019

4. Bates D, Mächler M, Bolker B, et al: Fitting linear mixed-effects models using lme4. Journal of Statistical Software 67:1-48, 2015
